# Systematic Study on the Cytotoxic Potency of Commonly Used Dimeric Metal Precursors in Human Cancer Cell Lines

**DOI:** 10.1002/open.202200019

**Published:** 2022-02-24

**Authors:** Heiko Geisler, Sophia Harringer, Dominik Wenisch, Richard Urban, Michael A. Jakupec, Wolfgang Kandioller, Bernhard K. Keppler

**Affiliations:** ^1^ Institute of Inorganic Chemistry University of Vienna Waehringer Str. 42 1090 Vienna Austria; ^2^ Research Cluster “Translational Cancer Therapy Research” Waehringer Str. 42 1090 Vienna Austria

**Keywords:** anticancer, inhibitory concentration, MTT assay, organometallic, piano stool

## Abstract

The cytotoxicities of seven dimeric metal species of the general formula [M(arene)Cl_2_]_2_, commonly used as precursors for complex synthesis and deemed biologically inactive, are investigated in seven commonly employed human cancer cell lines. Four of these complexes featured a ruthenium(II) core, where *p*‐cymene, toluene, benzene and indane were used as arenes. Furthermore, the osmium(II) *p*‐cymene dimer, as well as the Cp* dimers of rhodium(III) and its heavier analogue iridium(III) were included in this work (Cp*=1,2,3,4,5‐pentamethylcyclopentadienide). While the cytotoxic potencies of the ruthenium(II) and osmium(II) dimers are very low (or not even detectable at applicable concentrations), surprising activity, especially in cells from ovarian malignancies (with one or two‐digit micromolar IC_50_ values), have been found for the rhodium(III) and iridium(III) representatives. This publication is aimed at all researchers using synthetic procedures based on functionalization of these dimeric starting materials to rationalize changes in biological properties, especially cytotoxicity in cancer cells.

## Graphical abstract

**Dimeric metal precursors** are commonly employed in organometallic chemistry; however, comprehensive evaluation of their cytotoxic potency has been neglected so far. While organoruthenium dimers did not show appreciable cytotoxicity against seven commonly investigated human cancer cell lines, rhodium(III) and iridium(III) Cp* dimers exhibited notable activity in *in vitro* experiments.

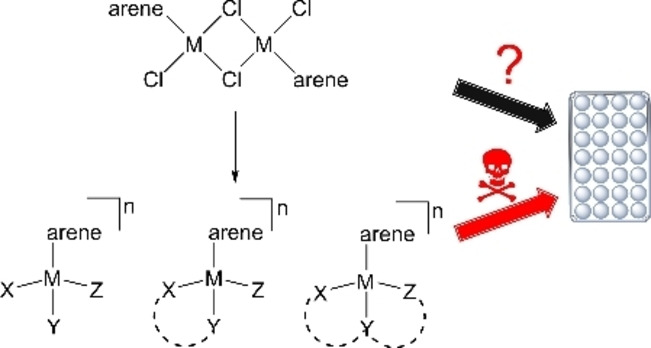

## Introduction

**Figure 1:**
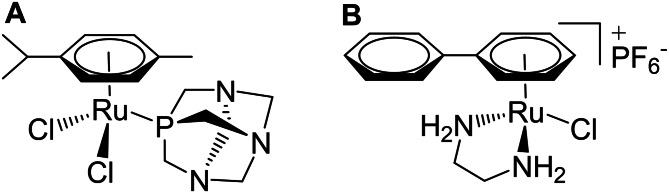
The structures of two well‐studied piano stool complexes, RAPTA‐C (**A**) and RM175 (**B**).

A wide range of different metal‐based drug candidates have gained interest as alternatives to classic platinum(II) anticancer agents.^[1]^ Historically, pioneer ruthenium complexes, structurally derived from cisplatin, were evaluated for their anticancer potency by M. J. Clarke et al. in the 1980s.^[2]^ In later years, BOLD‐100 (formerly KP1339, NKP‐1339, IT‐139) and NAMI‐A showed promising activity profiles in preclinical and clinical studies.^[3]^ From further investigations, the so‐called piano‐stool complexes emerged as a viable class of antitumor ruthenium compounds. These organometallics are composed of an arene moiety, stabilizing the metal center in its active oxidation state, as well as of mono‐, bi‐ or tri‐dentate ligands, constituting the stool's legs.^[4]^ Amongst the many advantages of working with these compounds, the easy modification of their pharmacokinetic and pharmacodynamic parameters through ligand variation is one of the most important.^[5]^ This enabled the fast generation of different series and libraries of piano‐stool complexes, which have been characterized and evaluated *in vitro* and *in vivo* (Figure 1).^[6]^

Many synthetic procedures are based on dimeric organometallic precursors by reaction with the ligands of choice, such as those developed by Dyson's,[^6e^, ^7^] and Sadler's group,[^6f^, ^8^] Süss‐Fink's trithiolato diruthenium compounds,^[9]^ or those from Hartinger's,[^6d^, ^10^] Marchetti's,^[11]^ Turel's^[12]^ as well as Therrien's lab,^[13]^ and several works from the authors of this publication.^[14]^

It is advisable that the biological investigation of new complexes includes a comparison with the individual building blocks to differentiate between biological properties that are already inherent to the latter and those arising from their incorporation into the final product. Knowledge of the biological properties of building blocks and starting materials as reference compounds is essential for assessing the potential benefits of the final product. It is already common practice to determine the cytotoxic potencies of the free ligands, even if they are not *a priori* expected to be biologically active themselves.^[6c]^ However, the dimeric precursors for the synthesis of many organometallic complexes have not been sufficiently evaluated for their *in vitro* cytotoxicity in our opinion. So far, most publications are based on the assumption that the dimeric metal precursors show no relevant anticancer activity; however, there are also examples where remarkable IC_50_ values were observed (e. g., in MCF‐7 cells).^[15]^ Overall, systematic studies where the IC_50_ values were determined, are scarce, and finding the respective values is laborious (Table 1). Another issue becomes apparent when considering incubation times, which lack standardization, and upper limits of tested concentration ranges, thus impeding comparison.

**Table 1:** Cytotoxicity values of dimeric metal precursors reported in the literature. IC_50_ values (in μm) in commonly employed human cancer cell lines. Values are means±SDs obtained from the respective assay (with exposure times given in the footnotes).

IC_50_ [μm]			
Cancer cell line	[RuCl_2_(*p*‐cym)]_2_ (**1**)	[RhCl_2_(Cp*)]_2_ (**6**)	[IrCl_2_(Cp*)]_2_ (**7**)
MCF‐7	5.27^[b,f,g]^ ^[15]^ >25^[b,g]^ ^[16]^ >100^[e,i,g]^ ^[17]^ 184±3^[d,g]^ ^[18]^		100±2^[d,g]^ ^[18]^
HeLa	>50^[a,g]^ ^[19]^		>50^[a,g]^ ^[19]^
A549	>50^[a,g]^ ^[19]^		>50^[a,g]^ ^[19]^
HT‐29	198±5^[d,g]^ ^[18]^		92±4^[d,g]^ ^[18]^
A2780	–	95±2^[d,g]^ ^[20]^	30.9±0.4^[d,g]^ ^[20]^
HL‐60	400.86±46.22^[b,g]^ ^[21]^		
NALM‐6	378.89±40.78^[b,g]^ ^[21]^		
WM‐115	>1000^[b,g]^ ^[21]^		
MDA‐MB‐453	>25^[b,g]^ ^[16]^		
SW480	>25^[b,g]^ ^[16]^		
IM9	>25^[b,g]^ ^[16]^		
PC3	213±6.90^[c,g]^ ^[22]^		
HT‐29	>100^[e,i]^ ^[17]^		
B16	>100^[a,j]^ ^[23]^		
C6	>100^[a,j]^ ^[23]^		
L929	>100^[a,j]^ ^[23]^		
HL‐60	>100^[a,j]^ ^[23]^		
K562	>100^[a,j]^ ^[23]^		
REH	>100^[a,j]^ ^[23]^		
HCT‐116	433±28^[c,h]^ ^[24]^		
NCI−H460	441±46^[c,h]^ ^[24]^		
SiHa	394±70^[c,h]^ ^[24]^		
SW480	346±48^[c,h]^ ^[25]^

Exposure time: [a] 24 h, [b] 48 h, [c] 72 h, [d] 5 days, [e] no exposure time given; [f] no±SD given; [g] MTT‐assay, [h] sulforhodamine B assay, [i] crystal violet assay, [j] acid phosphatase assay.

We wanted to provide the scientific community with a summary of IC_50_ values in a panel of commonly employed cancer cell lines. This communication is directed at all transition metal chemists searching for a source for the anticancer activity of their dimeric metal precursors. For this purpose, seven compounds of the general formula [M(arene)Cl_2_]_2_ have been synthesized, and their IC_50_ values have been determined in a panel of seven different human cancer cell lines by means of the MTT assay.

## Results and Discussion

**Scheme 1:**
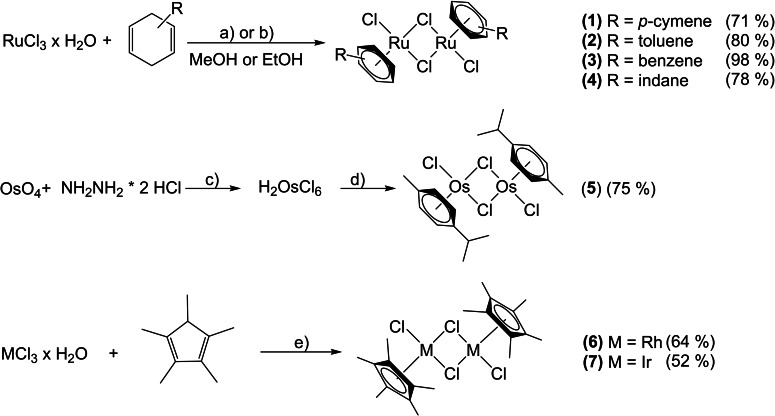
Reaction scheme of dimer syntheses (**1**–**7**): a)=reflux, 4‐26 h; b)=microwave, 120 °C, 3 min; c)=room temperature, 14 days; d)=reflux, 6 days; e)=reflux, 24–48 h.

The organometallic dimers **1**–**7** were synthesized according to literature (syntheses and minor modifications are described in the Supporting Information).^[26]^ The ruthenium, rhodium and iridium dimers (**1**–**4**, **6**, **7**) were synthesized by the treatment of the corresponding metal chlorides (MCl_3_; M=Ru, Rh, Ir) with dienes (α‐terpinene, cyclohexa‐1,4‐diene, 1‐methylcyclohexa‐1,4‐diene, 2,3,4,7‐tetrahydro‐1*H*‐indene, 1,2,3,4,5‐pentamethylcyclopentadiene=Cp*H), which provided good to excellent yields (52–98 %) (Scheme 1). OsO_4_ was treated with hydrazine dihydrochloride, yielding H_2_OsCl_6_, which was used without further purification. Afterwards, α‐terpinene was used as reducing agent to obtain the desired dimeric osmium(II) precursor (**5**) in good yield (75 %) over two steps (Scheme 1). Formation of the desired organometallic dimers was confirmed by NMR spectroscopy and the recorded shifts are in good agreement with reported literature data. Purity of the complexes was confirmed by elemental analysis.

**Figure 2:**
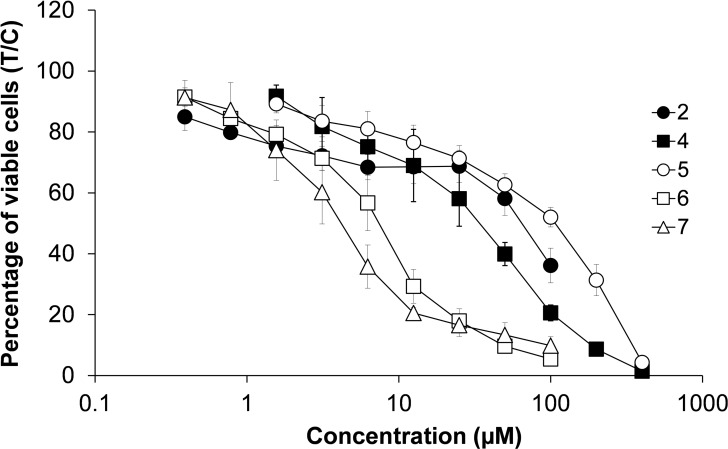
Concentration–effect curves of active compounds **2** and **4**–**7** in A2780 cells, obtained by the MTT assay (exposure time: 96 h). Values are means±SDs from at least three independent experiments.

To give the broadest overview in literature yet, the antiproliferative activity of all seven described dimeric precursors has been determined in exponentially growing monolayer cultures of seven adherent human cancer cell lines by means of the colorimetric MTT assay with 96 h exposure of cells to the compounds (plus 24 h tests to complement those settings which were studied further in the apoptosis/necrosis assay) (Table 2, Figure 2, Figure S8–S9; for experimental details, see the Supporting Information). Since many metal‐based compounds (including those firmly established in cancer chemotherapy) are rather slow‐acting due to their partial dependence on cell cycle progression, this long exposure time was preferred to avoid any potential cytotoxic activity going unnoticed. The chosen cell lines were A2780 (ovarian carcinoma), CH1/PA‐1 (ovarian teratocarcinoma), MCF‐7 (breast ductal carcinoma), A549 (lung adenocarcinoma), HCT‐116, HT29 and SW480 (all colon carcinoma). While the IC_50_ values of some complexes were determined precisely from concentration–effects curves even though a high micromolar range was required (e. g., **4**, **5**), others can only be given as >100 μm (**1**–**3**). These differences arise due to biophysical factors, mainly limited solubility.

**Table 2:** Cytotoxicity of dimeric metal precursors (**1**–**7**). IC_50_ values in seven human carcinoma cell lines. Values are means±SDs obtained by the MTT assay (exposure time: 96 h unless stated otherwise).

	IC_50_ [μm]							
	A2780, 24 h	A2780, 96 h	CH1/PA‐1	MCF‐7	A549	HCT‐116	HT29	SW480
**1**	n.d.	>100	>100	>100	>100	>100	>100	>100
**2**	>100	65±12	>100	>100	>100	>100	>100	>100
**3**	n.d.	>100	>100	>100	>100	>100	>100	>100
**4**	306±56	33±8	156±43	208±9	223±29	314±35	303±43	215±32
**5**	264±25	108±13	51±13	251±19	257±66	229±12	229±53	166±35
**6**	267±10	7.3±1.5	73±6	237±11	81±14	162±3	129±2	127±8
**7**	>400	4.2±1.1	29±1	279±43	47±14	205±33	142±5	202±28

For the dimeric ruthenium compounds (**1**–**4**), the results constitute a proof of principle, as these complexes show IC_50_ values higher than 100 μm in almost all of the investigated cell lines. The only exceptions to this trend were observed in A2780 cells where ruthenium benzene and indane dimers **2** and **4** showed IC_50_ values of 65 and 33 μm after 96 h, respectively. A similar pattern was observed for osmium *p*‐cymene complex **5**, which can be considered inactive in most of the cell lines, except for CH1/PA‐1 teratocarcinoma cells, where activity was observed in concentrations around 50 μm.

In the case of rhodium (**6**) and iridium (**7**) Cp* dimers, unexpected observations can be reported, as they showed moderate cytotoxicity in A549 and CH1/PA‐1 cells and turned out to be highly active in A2780 cells at an exposure time of 96 h (Figure 2).

In the four cell lines where these two dimers showed low potency (high IC_50_ values), rhodium compound **6** was more active, whereas this trend was reversed in the more sensitive cells. In other words, iridium dimer **7** was more active in A549, CH1/PA‐1, and A2780 cells compared to rhodium **6**. Their surprisingly high activity may be attributed to the slower ligand exchange rates,^[27]^ which might also explain why 24 h exposure is insufficient for any relevant and superior cytotoxicity. In the case of the ruthenium precursors, hydrolysis rates are comparably fast; thus, inactivation steps (e. g., formation of hydroxy‐bridged dimers, amino acid interactions) may take place considerably faster than for their rhodium and iridium counterparts. In an attempt to study apoptosis and necrosis induction in A2780 cells, no values markedly exceeding those of negative controls were found for **1**–**5** and **7** after 24 h of treatment. Only compound **6** induced necrosis in up to 9 % of the cells, depending on the concentration (Figure S10).

In this context, ruthenium complexes are an excellent example where the dimeric precursors lack activity, while their final products often show activities in the low micromolar range.^[28]^ In contrast, it is possible that some of the reported cytotoxicities of organorhodium or ‐iridium complexes may derive from the metal arene moiety. However, the other building blocks of the ligand scaffolds have a more pronounced impact on pharmacokinetics and pharmacodynamics.

## Conclusion

Seven commonly used dimeric metal precursors (**1**–**7**) were synthesized and their purity was confirmed by standard analytical methods (NMR spectroscopy and elemental analysis). The half maximal inhibitory activity of all compounds was determined in seven frequently investigated human cancer cell lines. The *p*‐cymene, toluene, and benzene organoruthenium dimers (**1**–**3**) were almost inactive in all tested cancer cell lines, while organorhodium and its heavier homologic iridium compound (**6**, **7**) revealed noteworthy cytotoxicity in several cell lines, especially in A2780. These findings showed that rhodium(III) and iridium(III) Cp* dimer exhibited notable activity in *in vitro* experiments and should be considered in future studies when the cytotoxicity of organorhodium and organoiridium metallodrugs are evaluated.

## Conflict of interest

The authors declare no conflict of interest.

1

## Supporting information

As a service to our authors and readers, this journal provides supporting information supplied by the authors. Such materials are peer reviewed and may be re‐organized for online delivery, but are not copy‐edited or typeset. Technical support issues arising from supporting information (other than missing files) should be addressed to the authors.

Supporting Information

## Data Availability

The data that support the findings of this study are available from the corresponding author upon reasonable request.

## References

[1] G. Gasser , I. Ott , N. Metzler-Nolte , J. Med. Chem. 2011, 54, 3–25. 21077686 10.1021/jm100020w PMC3018145

[2a] M. J. Clarke , Coord. Chem. Rev. 2002, 232, 69–93;

[2b] G. Suss-Fink , Dalton Trans. 2010, 39, 1673–1688. 20449402 10.1039/b916860p

[3a] E. Alessio , L. Messori , Molecules 2019, 24, 1995; 10.3390/molecules24101995 PMC6571951 31137659

[3b] S. Leijen , S. A. Burgers , P. Baas , D. Pluim , M. Tibben , E. van Werkhoven , E. Alessio , G. Sava , J. H. Beijnen , J. H. Schellens , Invest. New Drugs 2015, 33, 201–214; 25344453 10.1007/s10637-014-0179-1

[3c] H. A. Burris , S. Bakewell , J. C. Bendell , J. Infante , S. F. Jones , D. R. Spigel , G. J. Weiss , R. K. Ramanathan , A. Ogden , D. Von Hoff , ESMO Open 2016, 1, e000154. 28848672 10.1136/esmoopen-2016-000154 PMC5548977

[4] Y. K. Yan , M. Melchart , A. Habtemariam , P. J. Sadler , Chem. Commun. 2005, 38, 4764–4776. 10.1039/b508531b 16193110

[5] R. Fernández , M. Melchart , A. Habtemariam , S. Parsons , P. J. Sadler , Chem. Eur. J. 2004, 10, 5173–5179. 15372674 10.1002/chem.200400640

[6a] C. S. Allardyce , P. J. Dyson , Dalton Trans. 2016, 45, 3201–3209; 26820398 10.1039/c5dt03919c

[6b] B. Englinger , C. Pirker , P. Heffeter , A. Terenzi , C. R. Kowol , B. K. Keppler , W. Berger , Chem. Rev. 2019, 119, 1519–1624; 30489072 10.1021/acs.chemrev.8b00396

[6c] C. G. Hartinger , N. Metzler-Nolte , P. J. Dyson , Organometallics 2012, 31, 5677–5685;

[6d] D. Truong , M. P. Sullivan , K. K. H. Tong , T. R. Steel , A. Prause , J. H. Lovett , J. W. Andersen , S. M. F. Jamieson , H. H. Harris , I. Ott , C. M. Weekley , K. Hummitzsch , T. Sohnel , M. Hanif , N. Metzler-Nolte , D. C. Goldstone , C. G. Hartinger , Inorg. Chem. 2020, 59, 3281–3289; 32073260 10.1021/acs.inorgchem.9b03640

[6e] C. Scolaro , A. Bergamo , L. Brescacin , R. Delfino , M. Cocchietto , G. Laurenczy , T. J. Geldbach , G. Sava , P. J. Dyson , J. Med. Chem. 2005, 48, 4161–4171; 15943488 10.1021/jm050015d

[6f] R. E. Aird , J. Cummings , A. A. Ritchie , M. Muir , R. E. Morris , H. Chen , P. J. Sadler , D. I. Jodrell , Br. J. Cancer 2002, 86, 1652–1657. 12085218 10.1038/sj.bjc.6600290 PMC2746580

[7a] R. Pettinari , F. Marchetti , F. Condello , C. Pettinari , G. Lupidi , R. Scopelliti , S. Mukhopadhyay , T. Riedel , P. J. Dyson , Organometallics 2014, 33, 3709–3715;

[7b] R. Pettinari , A. Petrini , F. Marchetti , C. Pettinari , T. Riedel , B. Therrien , P. J. Dyson , Eur. J. Inorg. Chem. 2017, 2017, 1800–1806. 10.1021/acs.inorgchem.7b02356 29053264

[8a] Y. Fu , M. J. Romero , A. Habtemariam , M. E. Snowden , L. Song , G. J. Clarkson , B. Qamar , A. M. Pizarro , P. R. Unwin , P. J. Sadler , Chem. Sci. 2012, 3, 2485–2494;

[8b] W.-Y. Zhang , H. E. Bridgewater , S. Banerjee , J. J. Soldevila-Barreda , G. J. Clarkson , H. Shi , C. Imberti , P. J. Sadler , Eur. J. Inorg. Chem. 2020, 2020, 1052–1060. 33776557 10.1002/ejic.201901055 PMC7610438

[9] F. Giannini , J. Furrer , G. Süss-Fink , C. M. Clavel , P. J. Dyson , J. Organomet. Chem. 2013, 744, 41–48.

[10a] S. Moon , M. Hanif , M. Kubanik , H. Holtkamp , T. Söhnel , S. M. F. Jamieson , C. G. Hartinger , ChemPlusChem 2015, 80, 231–236;

[10b] W. D. J. Tremlett , K. K. H. Tong , T. R. Steel , S. Movassaghi , M. Hanif , S. M. F. Jamieson , T. Söhnel , C. G. Hartinger , J. Inorg. Biochem. 2019, 199, 110768. 31357065 10.1016/j.jinorgbio.2019.110768

[11a] L. Biancalana , M. Gruchała , L. K. Batchelor , A. Błauż , A. Monti , G. Pampaloni , B. Rychlik , P. J. Dyson , F. Marchetti , Eur. J. Inorg. Chem. 2020, 2020, 1061–1072;

[11b] J. Palmucci , F. Marchetti , R. Pettinari , C. Pettinari , R. Scopelliti , T. Riedel , B. Therrien , A. Galindo , P. J. Dyson , Inorg. Chem. 2016, 55, 11770–11781. 27934319 10.1021/acs.inorgchem.6b01861

[12a] J. Kljun , I. E. León , Š. Peršič , J. F. Cadavid-Vargas , S. B. Etcheverry , W. He , Y. Bai , I. Turel , J. Inorg. Biochem. 2018, 186, 187–196; 29960150 10.1016/j.jinorgbio.2018.05.009

[12b] K. Traven , I. Turel , J. Koziskova , L. Bucinsky , J. Koaeisek , Acta Crystallogr. Sect. C 2018, 74, 683–689; 10.1107/S2053229618006654 29870003

[12c] M. Ursic , T. Lipec , A. Meden , I. Turel , Molecules 2017, 22, 326. 10.3390/molecules22020326 PMC6155601 28230756

[13a] W. Aboura , L. K. Batchelor , A. Garci , P. J. Dyson , B. Therrien , Inorg. Chim. Acta 2020, 501, 119265;

[13b] V. Mannancherril , B. Therrien , Inorg. Chem. 2018, 57, 3626–3633. 29271651 10.1021/acs.inorgchem.7b02668

[14a] C. A. Riedl , L. S. Flocke , M. Hejl , A. Roller , M. H. M. Klose , M. A. Jakupec , W. Kandioller , B. K. Keppler , Inorg. Chem. 2017, 56, 528–541; 27996251 10.1021/acs.inorgchem.6b02430

[14b] J. H. Kasser , W. Kandioller , C. G. Hartinger , A. A. Nazarov , V. B. Arion , P. J. Dyson , B. K. Keppler , J. Organomet. Chem. 2010, 695, 875–881.

[15] T. A. Khan , K. Bhar , R. Thirumoorthi , T. K. Roy , A. K. Sharma , New J. Chem. 2020, 44, 239–257.

[16] M. Krstić , S. P. Sovilj , S. Grgurić-Šipka , I. R. Evans , S. Borozan , J. F. Santibanez , Eur. J. Med. Chem. 2011, 46, 4168–4177. 21741132 10.1016/j.ejmech.2011.06.019

[17] L. Oehninger , M. Stefanopoulou , H. Alborzinia , J. Schur , S. Ludewig , K. Namikawa , A. Munoz-Castro , R. W. Koster , K. Baumann , S. Wolfl , W. S. Sheldrick , I. Ott , Dalton Trans. 2013, 42, 1657–1666. 23149817 10.1039/c2dt32319b

[18] S. J. Lucas , R. M. Lord , R. L. Wilson , R. M. Phillips , V. Sridharan , P. C. McGowan , Dalton Trans. 2012, 41, 13800–13802. 23015068 10.1039/c2dt32104a

[19] Q. Du , L. Guo , M. Tian , X. Ge , Y. Yang , X. Jian , Z. Xu , Z. Tian , Z. Liu , Organometallics 2018, 37, 2880–2889.

[20] Z. Almodares , S. J. Lucas , B. D. Crossley , A. M. Basri , C. M. Pask , A. J. Hebden , R. M. Phillips , P. C. McGowan , Inorg. Chem. 2014, 53, 727–736. 24397747 10.1021/ic401529u

[21] E. Namiecińska , B. Sadowska , M. Więckowska-Szakiel , A. Dołęga , B. Pasternak , M. Grazul , E. Budzisz , RSC Adv. 2019, 9, 38629–38645. 35540189 10.1039/c9ra08736b PMC9075995

[22] Y. Benabdelouahab , L. Muñoz-Moreno , M. Frik , I. de la Cueva-Alique , M. A. El Amrani , M. Contel , A. M. Bajo , T. Cuenca , E. Royo , Eur. J. Inorg. Chem. 2015, 2015, 2295–2307. 27175101 10.1002/ejic.201500097 PMC4862618

[23] A. Savić , M. Dulović , J. M. Poljarević , S. Misirlić-Denčić , M. Jovanović , A. Bogdanović , V. Trajković , T. J. Sabo , S. Grgurić-Šipka , I. Marković , ChemMedChem 2011, 6, 1884–1891. 21805645 10.1002/cmdc.201100232

[24] A. Ashraf , M. Kubanik , F. Aman , H. Holtkamp , T. Söhnel , S. M. F. Jamieson , M. Hanif , W. A. Siddiqui , C. G. Hartinger , Eur. J. Inorg. Chem. 2016, 2016, 1376–1382.

[25] A. Ashraf , F. Aman , S. Movassaghi , A. Zafar , M. Kubanik , W. A. Siddiqui , J. Reynisson , T. Söhnel , S. M. F. Jamieson , M. Hanif , C. G. Hartinger , Organometallics 2019, 38, 361–374.

[26a] D. R. Baghurst , D. M. P. Mingos , J. Organomet. Chem. 1990, 384, C57–C60;

[26b] S. B. Jensen , S. J. Rodger , M. D. Spicer , J. Organomet. Chem. 1998, 556, 151–158;

[26c] M. A. Bennett , A. K. Smith , J. Chem. Soc. Dalton Trans. 1974, 2, 233–241;

[26d] J. W. Kang , K. Moseley , P. M. Maitlis , J. Am. Chem. Soc. 1969, 91, 5970–5977;

[26e] W. A. Kiel , R. G. Ball , W. A. G. Graham , J. Organomet. Chem. 1990, 383, 481–496;

[26f] L. Ma , R. Ma , Z. Wang , S.-M. Yiu , G. Zhu , Chem. Commun. 2016, 52, 10735–10738. 10.1039/c6cc04354b 27506281

[27a] L. Helm , A. E. Merbach , J. Chem. Soc. Dalton Trans. 2002, 5, 633–641;

[27b] P. C. A. Bruijnincx , P. J. Sadler , in Advances in Inorganic Chemistry, Vol. 61 (Eds.: R. van Eldik , C. D. Hubbard ), Academic Press, 2009, pp. 1–62. 10.1016/S0898-8838(09)00201-3 PMC3024542 21258628

[28a] J. Furrer , G. Süss-Fink , Coord. Chem. Rev. 2016, 309, 36–50;

[28b] H. Geisler , D. Wernitznig , M. Hejl , N. Gajic , M. A. Jakupec , W. Kandioller , B. K. Keppler , Dalton Trans. 2020, 49, 1393–1397. 31950944 10.1039/c9dt04462k

