# Supplementary material for: Systematic Study on the Cytotoxic Potency of Commonly Used Dimeric Metal Precursors in Human Cancer Cell Lines
Source: ChemistryOpen. 2022 Feb 24;11(7):e202200019. doi: 10.1002/open.202200019 (PMC9278098; doi:10.1002/open.202200019)
Supplement: Supplementary file 1 — Supporting Information [file OPEN-11-e202200019-s001.pdf]

ChemistryOpen

Supporting Information

Systematic Study on the Cytotoxic Potency of Commonly Used Dimeric Metal Precursors in Human Cancer Cell Lines

Heiko Geisler[+], Sophia Harringer[+], Dominik Wenisch, Richard Urban, Michael A. Jakupec,* Wolfgang Kandioller,* and Bernhard K. Keppler

Contents

Experimental details S1

Material and methods S1

Dimeric metal compound syntheses (1–7) S1

Cell culture S3

Cytotoxicity test S3

Induction of apoptosis/necrosis S4

^{1}H-NMR spectra S5

MTT assay S9

Apoptosis assay S12

References S14

Experimental details

Material and methods

All dimeric compounds (**1–7**) [1] and 2,3,4,7 tetrahydro 1*H*-indene[2] were synthesized according to literature with minor modifications in the synthesis of **1–5**. The modifications are described in the experimental part where applicable. Solvents (methanol, ethanol, *n*-hexane, HCl (37%) and diethyl ether) were purchased from commercial sources and dried before use if needed. $RuCl_3$ •x H_2O, $RhCl_3$ •x H_2O, $IrCl_3$ •x H_2O and OsO_4 were purchased from Johnson Matthey, 1-methyl-1,4-cyclohexadiene and α-terpinene from Alfa Aesar, hydrazine dihydrochloride and cyclohexa 1,3-diene from Sigma-Aldrich, and 1,2,3,4,5-pentamethylcyclopentadiene from TCI Europe. Microwave reactions were performed with a Biotage® Initiator+ system. ^1H spectra were recorded at 298 K on a Bruker AV NEO 500 spectrometer at 500.10 MHz. Elemental analysis of the dimeric metal compounds (**1–7**) were conducted by microanalytical laboratory of the faculty of chemistry of the University of Vienna with a Perkin Elmer 2400 CHN elemental analyzer.

Dimeric metal compound syntheses (1–7)

Bis[dichlorido(η^6-*p*-cymene)ruthenium(II)] (**1**) [1c]: $RuCl_3$ x H_2O (1.95 g, 9.38 mmol) was dissolved in 60 mL methanol. α-Terpinene (12.50 g, 92.15 mmol) was added and the mixture was heated under reflux for 4 h. Afterwards, 20 mL diethyl ether was added to complete precipitation. The red solid was separated, washed with diethyl ether and dried *in vacuo*. Yield: 2.05 g; red solid (71%). ^1H NMR (500.10 MHz, $CDCl_3$) δ 5.47 (d, J = 6.0 Hz, 4H), 5.34 (d, J = 6.0 Hz, 4H), 2.92 (hept, J = 7.0 Hz, 2H), 2.16 (s, 6H), 1.27 (d, J = 6.9 Hz, 6H). Anal. Calc. for $C_{20}H_{28}Ru_2Cl_4$: C 39.23%, H 4.61%. Found: C: 39.11%, H 4.54%.

Bis[dichlorido(η^6-toluene)ruthenium(II)] (**2**) [1f]: $RuCl_3$ x H_2O (1.00 g, 4.62 mmol) and 1-methyl-1,4-cyclohexadiene (4.35 g, 46.20 mmol) were dissolved in 40 mL ethanol and heated to reflux for 4 hours. The mixture was stored at 4 °C overnight. Afterwards, the red solid was separated, washed with cold ethanol and diethyl ether and dried *in vacuo*. Yield: 0.98 g; red solid (80%). ^1H NMR (500.10 MHz, DMSO-d_6) δ 5.99 (dd, J = 6.5, 5.4 Hz, 1H), 5.72–5.68 (m, 3H), 2.14 (s, 3H). Anal. Calc. for $C_{14}H_{16}Ru_2Cl_4$: C 31.83%, H 3.05%. Found: C: 31.59%, H 3.01%.

Bis[dichlorido(η^6-benzene)ruthenium(II)] (**3**) [1a]: $RuCl_3$ x H_2O (1.00 g, 3.86 mmol) and cyclohexa-1,3-diene (1.55 g, 19.30 mmol) were dissolved in 10 mL ethanol and stirred under microwave irradiation at 120 °C for three minutes. The solid was separated, washed with 100 mL cold ethanol and 50 mL diethyl ether and dried *in vacuo*. Yield: 0.96 g; red solid (98%). ^{1}H NMR (500.10 MHz, DMSO-d_6) δ 5.97 (s, 12H). Anal. Calc. for $C_{12}H_{12}Ru_2Cl_4$: C 28.81%, H 2.42%. Found: C: 28.82%, H 2.42%.

Bis[dichlorido(η^6-indane)ruthenium(II)] (**4**) [1b]: $RuCl_3$ x H_2O (343 mg, 1.65 mmol) and 2,3,4,7-tetrahydro-1H-indene (1395 mg, 11.60 mmol) were dissolved in 60 mL dry ethanol and refluxed for 26 h under argon atmosphere. After the mixture had cooled to room temperature, diethyl ether was added for complete precipitation. The red solid was filtered, washed with *n*-hexane and dried *in vacuo*. Yield: 376 mg; red solid (78%). ^{1}H NMR (500.10 MHz, $CDCl_3$) δ 5.61–5.58 (m, 4H), 5.56–5.53 (m, 4H), 2.97–2.85 (m, 4H), 2.43–2.29 (m, 6H), 2.09–1.94 (m, 2H). Anal. Calc. for $C_{18}H_{20}Ru_2Cl_4$: C 37.26%, H 3.47%. Found: C: 37.08%, H 3.49%.

Bis[dichlorido(η^6-*p*-cymene)osmium(II)] (**5**) [1e]: Hydrazine dihydrochloride (0.84 g, 8.00 mmol) was added to a suspension of OsO_4 (2 g, 7.87 mmol) in 50 mL HCl (37 %) and stirred for 14 days at room temperature under argon atmosphere. The mixture was filtered and washed with HCl (37%). The solvent was removed under reduced pressure affording a dark red oil. Afterwards, the oil was washed with 20 mL ethanol and the solvent was reduced under reduced pressure. This washing step was repeated three times. The red oil was dissolved in 40 mL ethanol and α-terpinene (8.37 g, 61.44 mmol) was added. The mixture was heated under reflux and light protection for 6 days. The orange precipitate was separated by filtration, washed with *n*-hexane and dried *in vacuo.* Yield: 2.42 g; orange solid (78%). ^{1}H NMR (500.10 MHz, $CDCl_3$) δ 6.18 (d, J = 5.5 Hz, 1H), 6.02 (d, J = 5.5 Hz, 1H), 2.78 (hept, J = 6.9 Hz, 1H), 2.20 (s, 2H), 1.29 (d, J = 7.0 Hz, 3H). Anal. Calc. for $C_{20}H_{28}Os_2Cl_4$: C 30.37%, H 3.57%. Found: C: 30.38%, H 3.82%.

Bis[dichlorido(η^5-1,2,3,4,5-pentamethylcyclopentadienyl)rhodium(III)] (**6**) [1d]: $RhCl_3$ x H_2O (1.50 g, 7.17 mmol) and 1,2,3,4,5-pentamethylcyclopentadiene (1.95 g, 14.30 mmol) were dissolved in 50 mL methanol and refluxed for 24 h. The mixture was cooled to room temperature and the red precipitate was separated by filtration, washed with diethyl ether and dried *in vacuo*. Yield: 1.41 g; red solid (64%). ^{1}H NMR (500.10 MHz,

$CDCl_3$) δ 1.60 (s, 15H). Anal. Calc. for $C_{20}H_{30}Rh_2Cl_4$: C 38.86%, H 4.89%. Found: C: 38.88%, H 5.07%.

Bis[dichlorido(η^5-1,2,3,4,5-pentamethylcyclopentadienyl)iridium(III)] (**7**) [1d]: $IrCl_3$ x H_2O (1.00 g, 3.35 mmol) and 1,2,3,4,5-pentamethylcyclopentadiene (0.91 g, 6.70 mmol) were dissolved in 40 mL methanol and refluxed for 48 h. The mixture was cooled to room temperature and the red precipitate was separated by filtration, washed with diethyl ether and dried *in vacuo*. Yield: 0.69 g; orange solid (52%). ^{1}H NMR (500.10 MHz, $CDCl_3$) δ 1.59 (s, 15H). Anal. Calc. for $C_{20}H_{30}Ir_2Cl_4 \cdot 0.5H_2O$: C 29.81%, H 3.88%. Found: C: 29.57%, H 3.84%.

Cell culture

CH1/PA-1 cells (identified *via* STR profiling as PA-1 ovarian teratocarcinoma cells by Multiplexion, Heidelberg, Germany) were a gift from Lloyd R. Kelland, CRC Center for Cancer Therapeutics, Institute of Cancer Research, Sutton, UK. SW480 (human adenocarcinoma of the colon) and A549 (human non-small cell lung cancer) cells were provided by the Institute of Cancer Research, Department of Medicine I, Medical University of Vienna, Austria. All cell culture media, supplements and reagents were purchased from Sigma-Aldrich, and plasticware from Starlab. Cells were grown in 75 cm^2 culture flasks as adherent cultures in minimum essential medium (MEM) supplemented with 10% heat-inactivated fetal bovine serum (BioWest),1 mM sodium pyruvate, 4 mM L-glutamine, and 1% non-essential amino acids (from 100× ready-to-use stock). Cultures were maintained at 37 °C in a humidified atmosphere composed of 95% air and 5% CO_2.

Cytotoxicity test

Cytotoxic effects were determined by means of the MTT-based colorimetric microculture assay. Cells were harvested from flasks by trypsinization and seeded in 100 mL supplemented MEM per well into 96-well flat-bottom microculture plates. Cell densities of 1.0×10^3 cells/well (CH1/PA-1), 2.0×10^3 cells/well (SW480), and 3.0×10^3 cells/well (A549) were chosen to ensure exponential growth of untreated controls throughout the experiment, and cells were allowed to resume adherent growth for 24 h. Test compounds were dissolved in DMSO, diluted in supplemented MEM and added to the plates where the final DMSO content did not exceed 0.5%. After 96 h of exposure, MEM was replaced with 100 mL/well of a 1:7 MTT/RPMI 1640 mixture (one part of 5 mg/mL MTT in

phosphate-buffered saline; six parts of RPMI 1640 medium supplemented with 10% heat-inactivated fetal bovine serum and 4 mM L-glutamine). After incubation for 4 h, the supernatant was removed and the formazan product formed by viable cells dissolved in 150 mL DMSO per well. Optical densities at 550 nm (and 690 nm as a reference) were measured with a BioTek ELx808 microplate reader and Gen5 software. Quantities of viable cells were expressed relative to untreated controls, and 50% inhibitory concentrations (IC_{50}) were interpolated from concentration-effect curves. At least three independent experiments were performed, with triplicates for each concentration level.

Induction of apoptosis/necrosis

Percentages of apoptotic and necrotic cells after exposure to test substances for 24 h were evaluated by flow cytometry. For this purpose, A2780 cells were harvested by trypsinization and seeded in 600 µL RPMI 1640 medium (supplemented as described above) per well into 24-well flat-bottom plates at a density of 7.0 x 10^4 cells/well. Cells were allowed to resume adherent growth for 24 h. Test substances were dissolved in DMSO, diluted in supplemented RPMI 1640 medium and added to the wells, where the final DMSO content did not exceed 0.5% v/v. After 24 h incubation, supernatants were separately collected in 5 mL Eppendorf tubes, cells were washed with PBS and added to the respective tube. Remaining cells were trypsinized for 2.5–5 min at 37 °C, and after inactivating trypsin with supplemented RPMI 1640 medium, cell suspensions were added to the respective tubes. The tubes were centrifuged at 2100 rpm for 3 min, supernatants confined, and cells were transferred into 1.5 mL Eppendorf tubes. Samples were centrifuged at 2100 rpm for 3 min, supernatants were aspirated completely, and annexin V-FITC solution (per sample: 1.5 µL human annexin V-FITC recombinant protein (Invitrogen) in 150 µL binding buffer: 10 mM HEPES/NaOH, pH 7.4; 140 mM NaCl, 2.5 mM $CaCl_2$ x $2H_2O$) were added. Samples were incubated for 15 min at 37 °C, upon which propidium iodide (PI) solution (per sample: 1 µL PI (Invitrogen; 1 mg/mL solution in water) in 150 µL binding buffer) were added. Samples were analyzed with a Millipore guava easyCyte 8HT flow cytometer. Data of at least three independent experiments were evaluated with FlowJo software.

^{1}H-NMR spectra

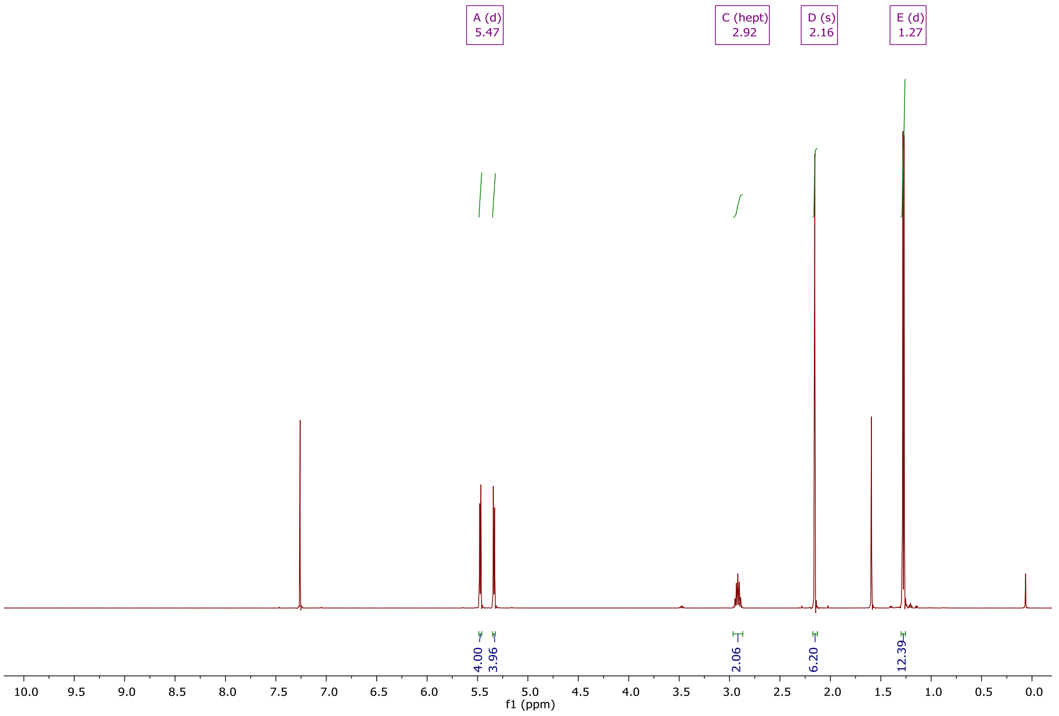

Figure S1: ^{1}H-NMR spectrum of compound **1**

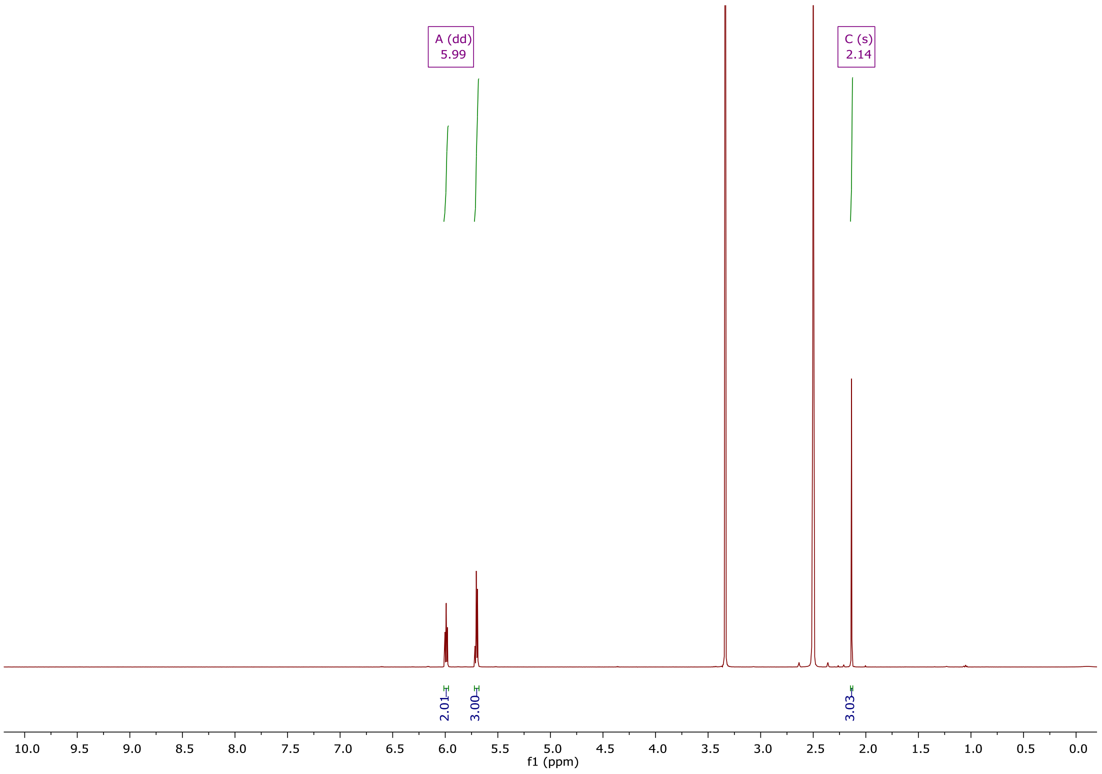

Figure S2: ^{1}H-NMR spectrum of compound **2**

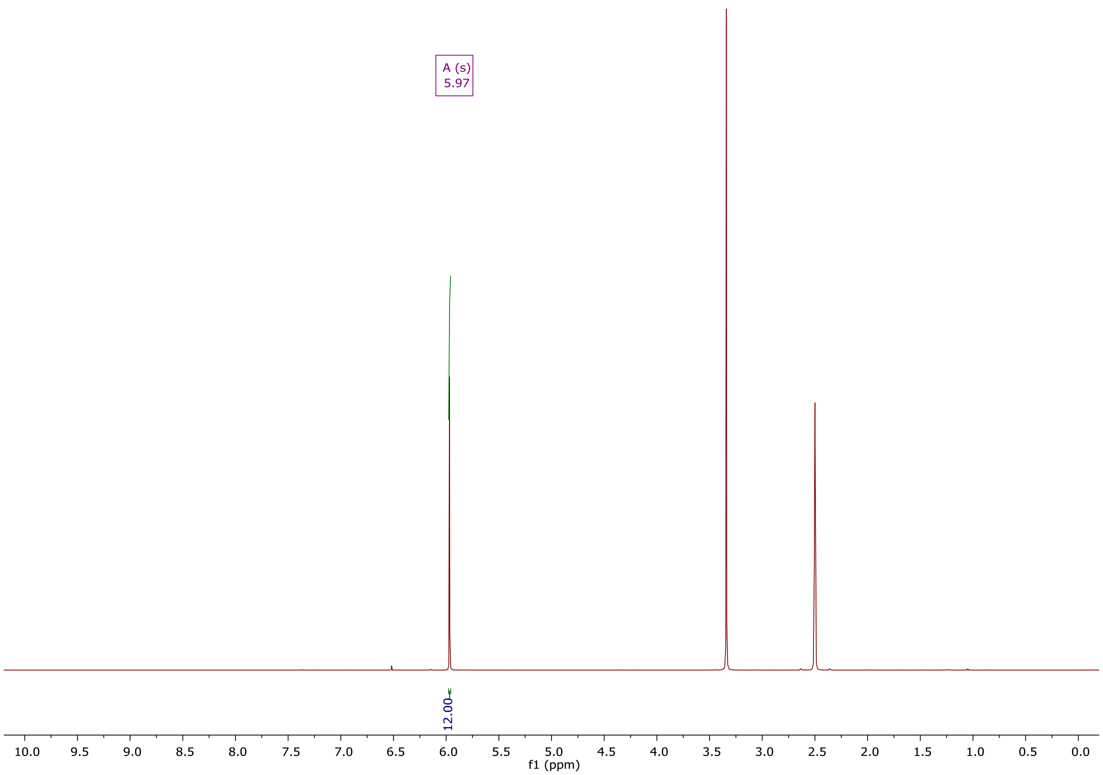

Figure S3: ^{1}H-NMR spectrum of compound **3**

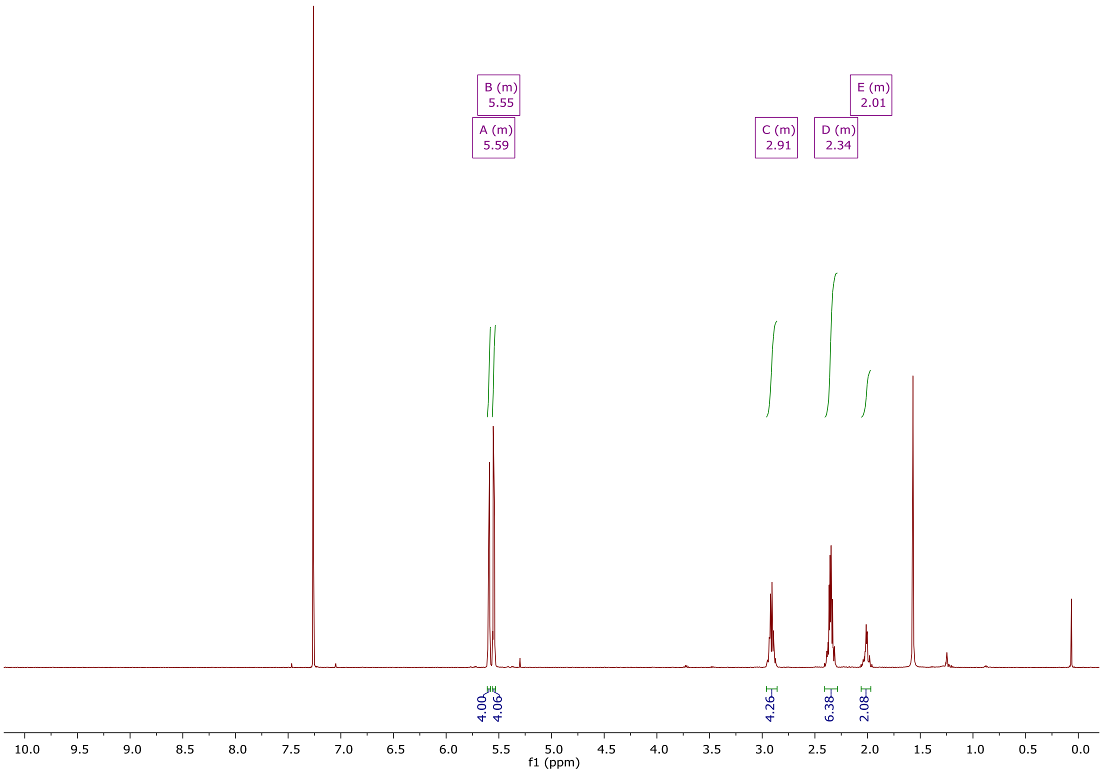

Figure S4: ^{1}H-NMR spectrum of compound **4**

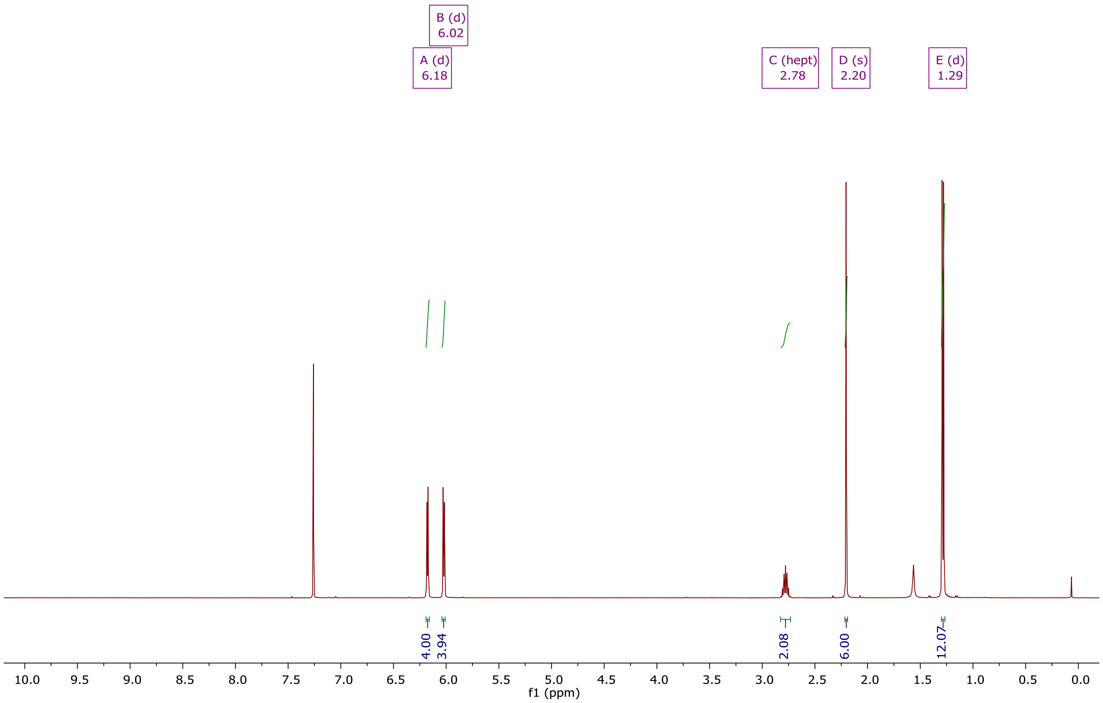

Figure S5: ^{1}H-NMR spectrum of compound **5**

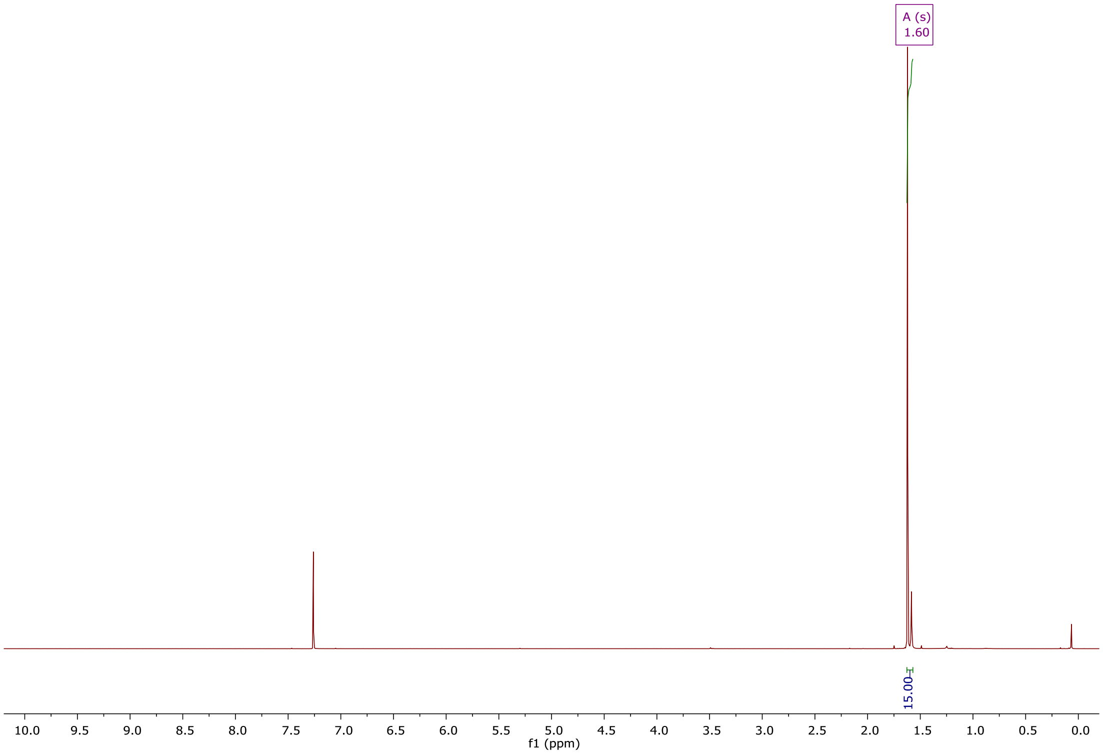

Figure S6: ^{1}H-NMR spectrum of compound **6**

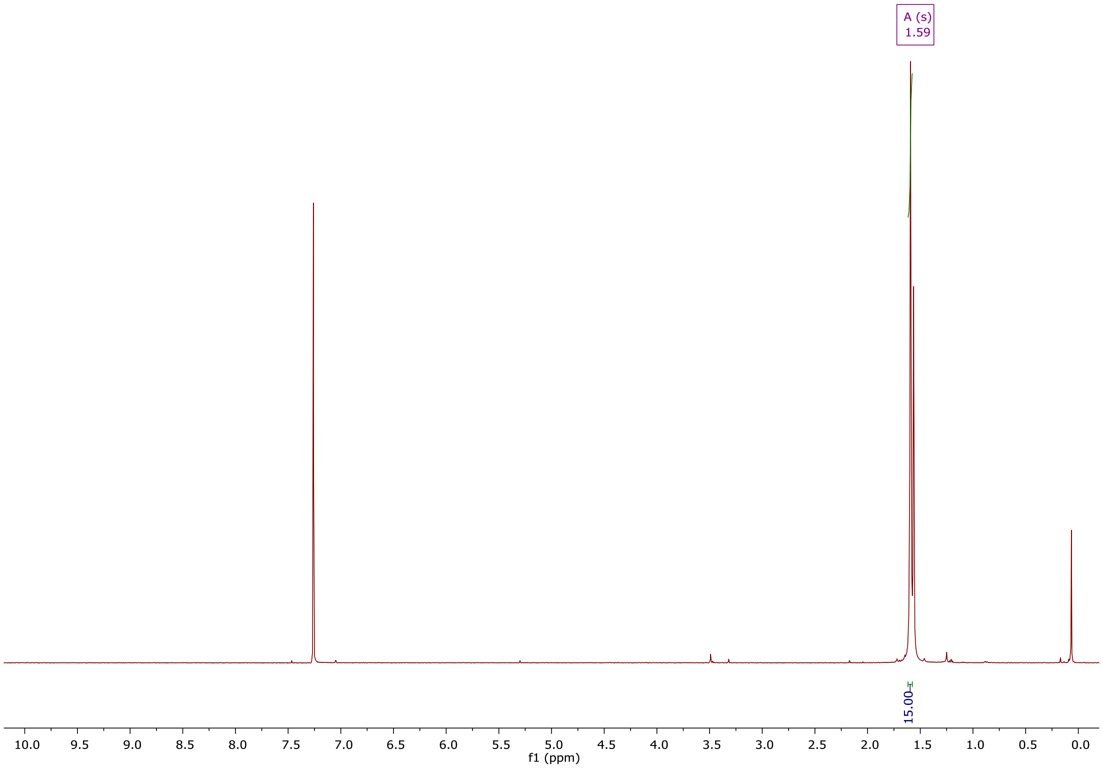

Figure S7: ^{1}H-NMR spectrum of compound **7**

MTT assay

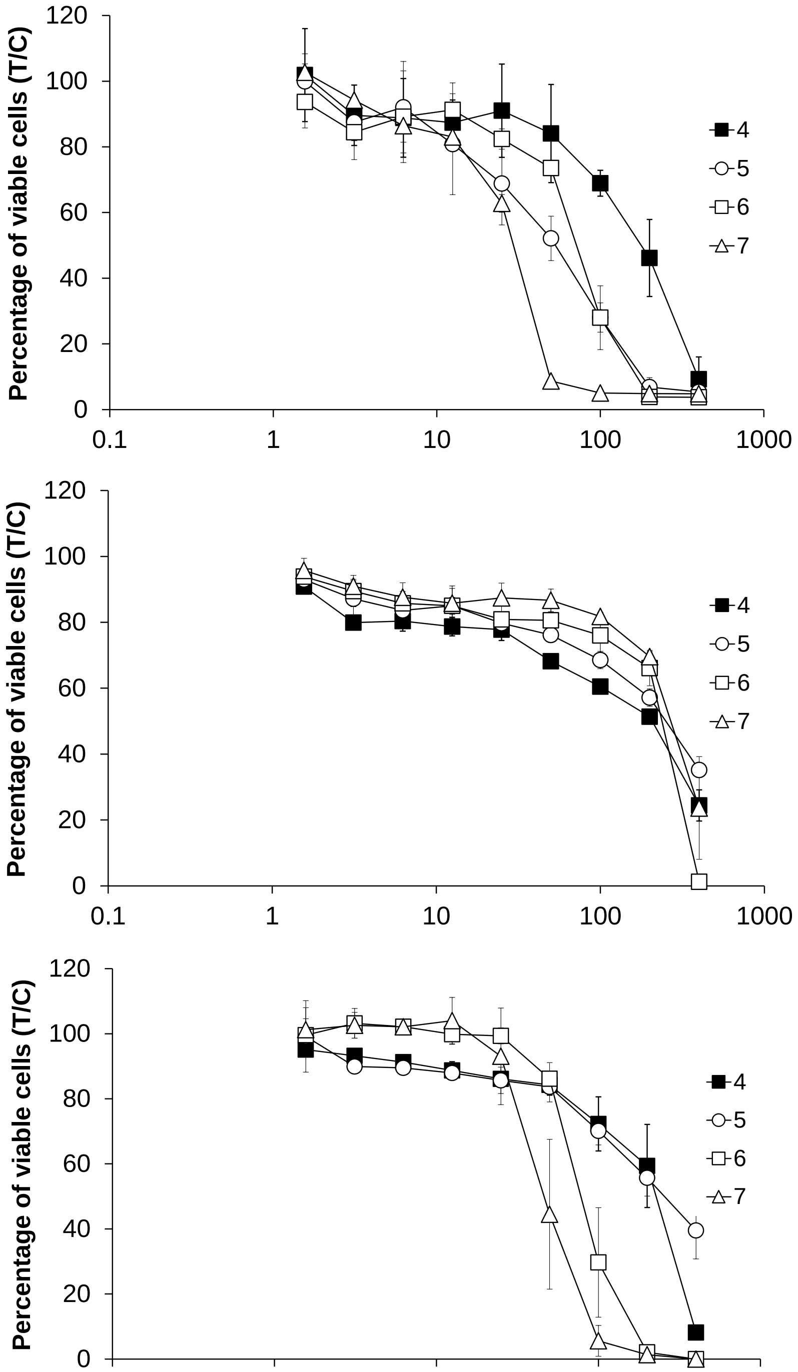

Figure S8. Concentration–effect curves of compounds **4**–**7** in CH1/PA-1 (top), MCF7 (middle) and A549 (bottom) cells, obtained by the MTT assay (exposure time: 96 h). Values are means ± SDs from at least three independent experiments.

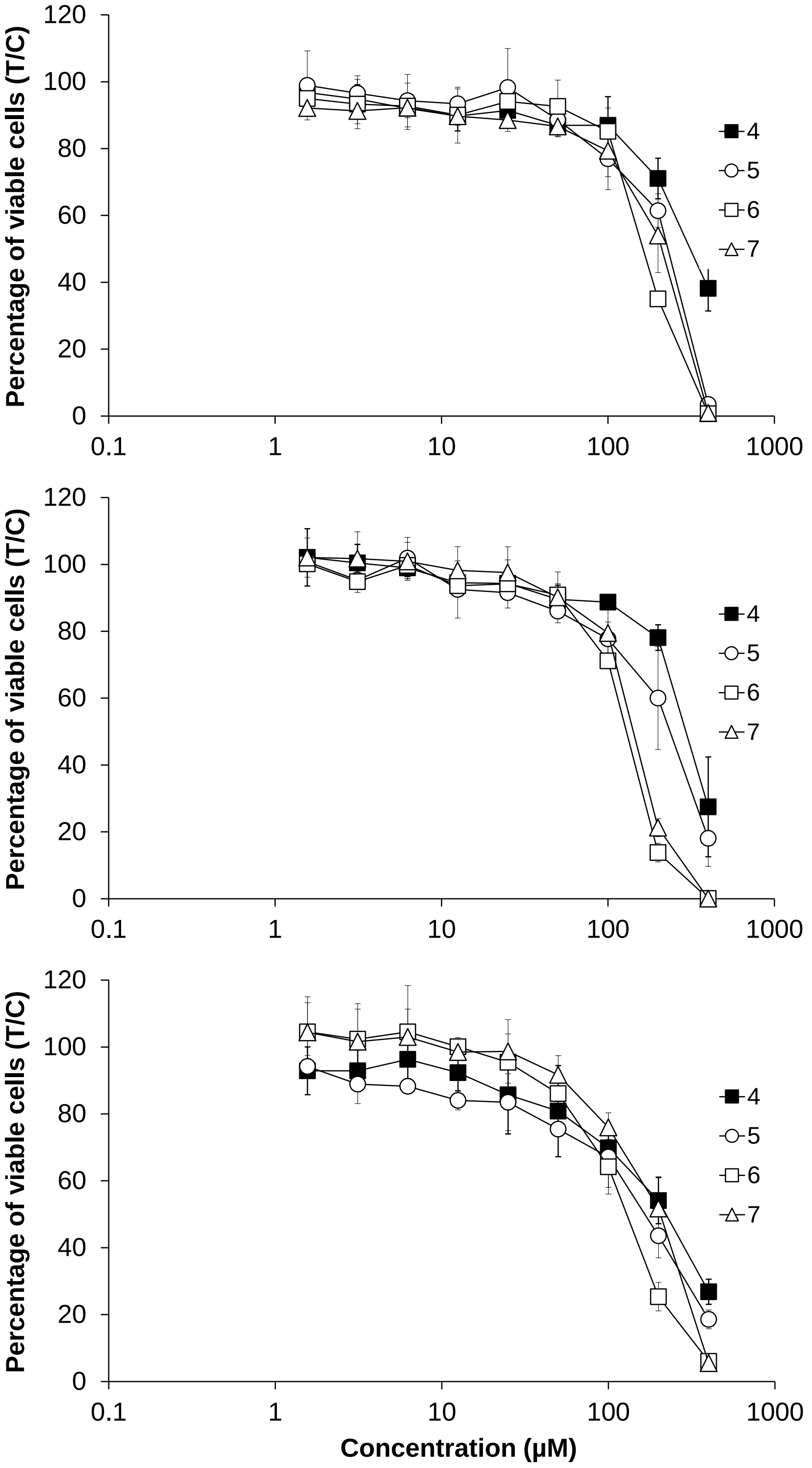

Figure S9. Concentration–effect curves of compounds **4**–**7** in HCT-116 (top), HT29 (middle) and SW480 (bottom) cells, obtained by the MTT assay (exposure time: 96 h). Values are means ± SDs from at least three independent experiments.

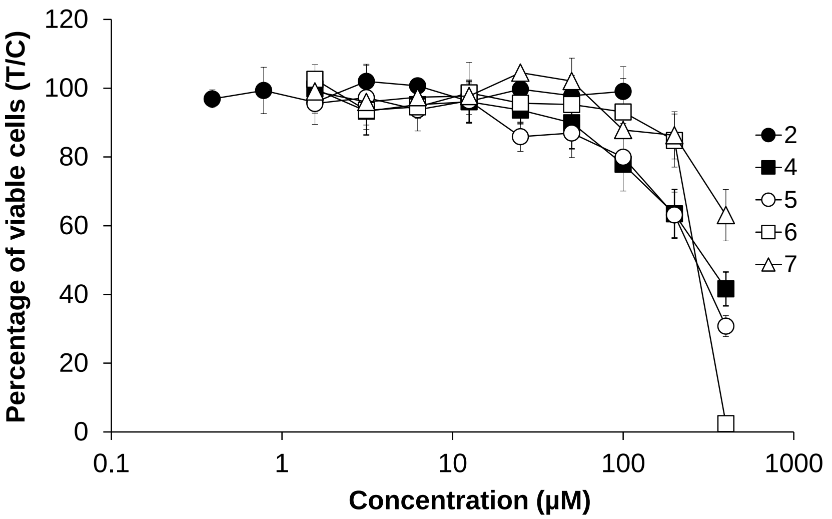

Figure S10. Concentration–effect curves of active compounds **2** and **4–7** in A2780 cells, obtained by the MTT assay (exposure time: 24 h). Values are means ± SDs from at least three independent experiments.

Apoptosis assay

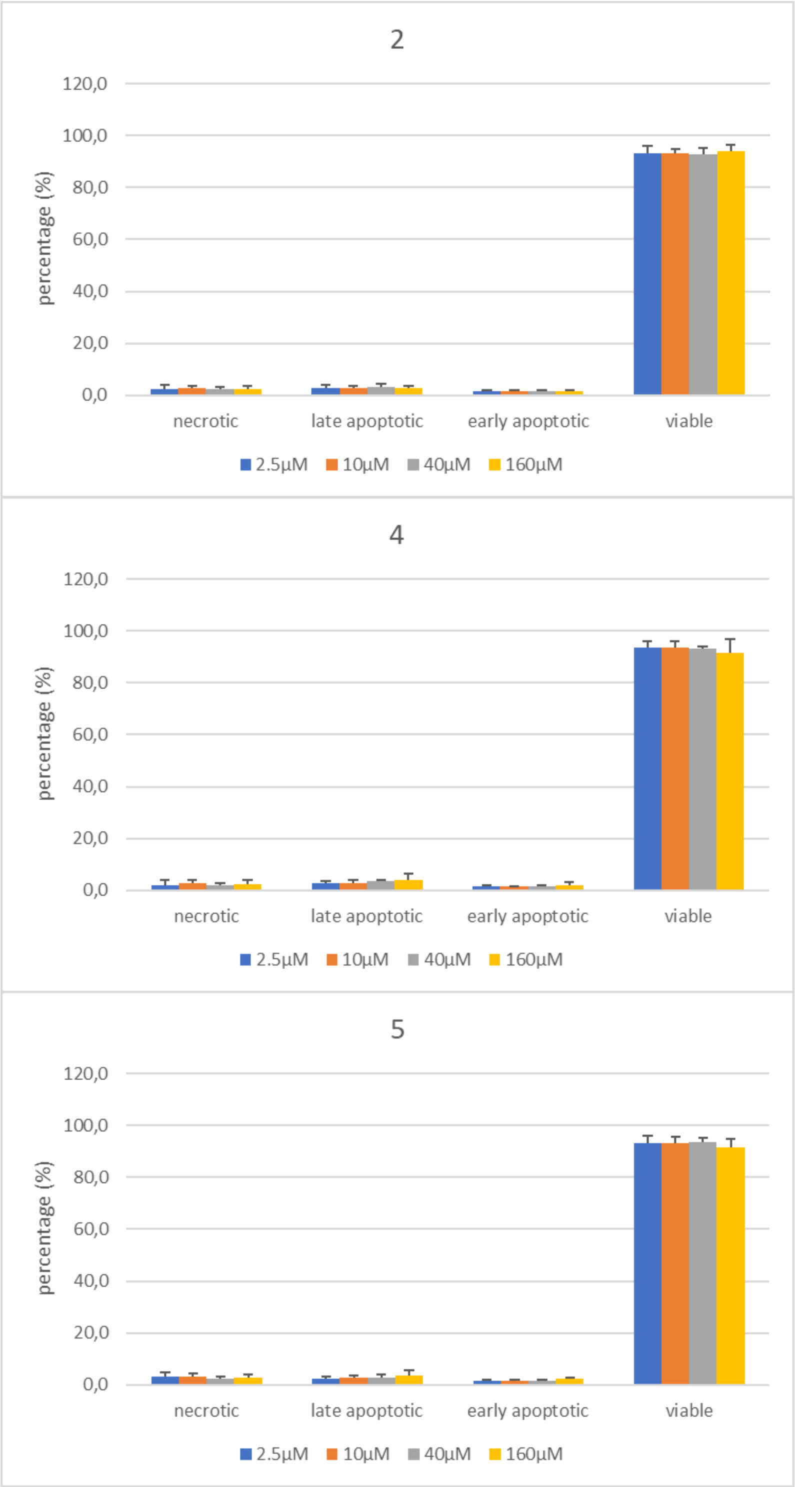

Figure S11. Concentration-dependent induction of apoptosis and necrosis by compounds **2** and **4–5** in A2780 cells upon exposure for 24h, evaluated by a flow-cytometric apoptosis assay using annexin V-FITC/propidium iodide double staining. Values are means ± SDs from at least three independent experiments.

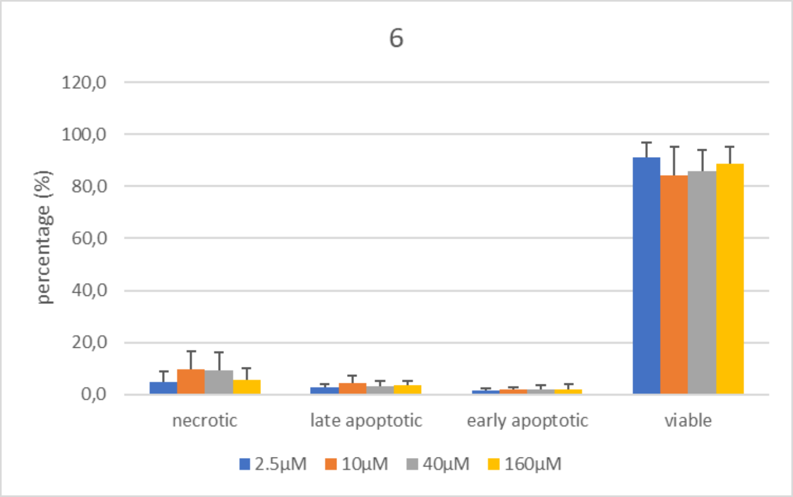

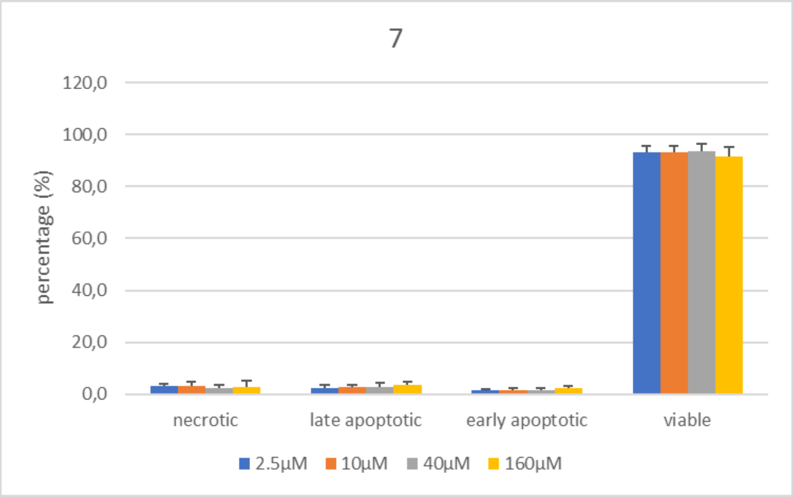

Figure S11 (continued).

References

[1] a) D. R. Baghurst, D. M. P. Mingos, *J. Organomet. Chem.* **1990**, *384*, C57-C60; b) M. A. Bennett, A. K. Smith, *J. Chem. Soc., Dalton Trans.* **1974**, 233-241; c) S. B. Jensen, S. J. Rodger, M. D. Spicer, *J. Organomet. Chem.* **1998**, *556*, 151-158; d) J. W. Kang, K. Moseley, P. M. Maitlis, *J. Am. Chem. Soc.* **1969**, *91*, 5970-5977; e) W. A. Kiel, R. G. Ball, W. A. G. Graham, *J. Organomet. Chem.* **1990**, *383*, 481-496; f) L. Ma, R. Ma, Z. Wang, S.-M. Yiu, G. Zhu, *Chem. Commun.* **2016**, *52*, 10735-10738.

[2] S. E. Condon, C. Buron, E. M. Tippmann, C. Tinner, M. S. Platz, *Org Lett* **2004**, *6*, 815-818.
